# The Efficacy of Tumor Characterization for Colorectal Lesions with Blue Light Imaging of a Compact Light-Emitting Diode Endoscopic System Compared to a Laser Endoscopic System: A Pilot Study

**DOI:** 10.1155/2022/9998280

**Published:** 2022-04-12

**Authors:** Ken Inoue, Naohisa Yoshida, Reo Kobayashi, Yuri Tomita, Hikaru Hashimoto, Satoshi Sugino, Ryohei Hirose, Osamu Dohi, Hiroaki Yasuda, Ritsu Yasuda, Takaaki Murakami, Yutaka Inada, Yoshito Itoh

**Affiliations:** ^1^Molecular Gastroenterology and Hepatology, Kyoto Prefectural University of Medicine Graduate School of Medical Science, Kyoto, Japan; ^2^Department of Gastroenterology, Nara City Hospital, Nara, Japan; ^3^Department of Gastroenterology, Aiseikai Yamashina Hospital, Kyoto, Japan; ^4^Department of Gastroenterology and Hepatology, Japanese Red Cross Society Kyoto Daiichi Hospital, Kyoto, Japan

## Abstract

*Background*: A compact and cost-effective light source-processor combined 3-color light-emitting diode (LED) endoscopic system (ELUXEO-Lite: EP-6000, Fujifilm Co., Tokyo) with a magnified colonoscope (EC-6600ZP, Fujifilm Co.) has been released. *Aims*: In this study, we analyzed the efficacy of this system for colorectal tumor characterization with magnified blue light imaging (BLI-LED) and image's subjective and objective evaluations, compared to a magnified blue laser imaging (BLI-LASER) using a standard LASER endoscopic system. *Methods*: We retrospectively reviewed 37 lesions observed with both BLI-LED and BLI-LASER systems from 2019 using the Japanese narrow band imaging classification. Two representative magnified images, one BLI-LED and one BLI-LASER, of the same area of a lesion were evaluated for diagnostic accuracy and visualization quality by three experts and three non-experts. Their color difference values (CDVs) and brightness values (BVs) were also calculated as objective indicators. *Results*: Among 37 lesions, mean tumor size was 18.9 ± 13.1 mm, and 21 lesions were nonpolypoid. Histopathology revealed 14 sessile serrated lesions, 7 adenomas, 12 high-grade dysplasias and T1a cancers, and 4 T1b cancers. The diagnostic accuracy rates of BLI-LED/BLI-LASER of experts and non-experts were 90.1% and 87.4% (*p* = 0.52) and 89.2% and 89.2% (*p* = 0.99). The percentages of instances where BLI-LED images were better, the two imaging types were equivalent, or BLI-LASER images were better were 16%/83%/1% for experts and 19%/58%/23% for non-experts (*p* < 0.001). CDVs and BVs between BLI-LED and BLI-LASER were not significantly different (CDVs: *p* = 0.653, BVs: *p* = 0.518). *Conclusions*: BLI-LED using the compact system was noninferior to BLI-LASER for colorectal tumor characterization and image quality.

## 1. Introduction

A LASER endoscopic system (LASEREO; LL-4450 and LL-7000, Fujifilm Co., Tokyo Japan), with two laser wavelengths of 410 nm and 450 nm, was developed in 2012 [1]. This system allows white light imaging (WLI), blue light laser imaging (BLI-LASER), and linked color imaging (LCI) [2–7]. BLI-LASER with magnification has been reported to be useful for tumor characterization including the capability for diagnosing diminutive polyps with more than 90% of negative predictive values [2, 8]. On the other hand, the first report about application of white LEDs to colonoscopy showed the use of an LED colonoscope in 2011 [9]. LED light endoscope (ELUXEO, BL-7000 and VP-7000; Fujifilm Co.) has been marketed since 2017 in the West [10]. Multilight technology generates white light and short-wavelength narrowband light for accurate diagnosis and detection by controlling the emission intensity ratio of four LED lights (blue-violet, blue, green, and red). LED endoscopy (EC-760R-VL and EC-760ZP-VL) provides bright high-quality images with WLI, blue light imaging (BLI-LED), and LCI [11–13]. Both of LCI with LED and LASER endoscopes could reduce the miss rate of neoplastic lesions compared to WLI [12, 14, 15]. In 2020, a novel LED endoscopic system (EVIS X1, Olympus Co. Tokyo, Japan) with five LED lights (violet, blue, green, amber, and red) has appeared on the market for improving tumor detection and tumor characterization [13].

Another unique LED system, a compact light source-processor combined with an LED endoscopic system (ELUXEO Lite EP-6000, Fujifilm Co.) has been marketed since 2018 in Japan and Europe. We expected that multilight technology of LED endoscope, with three LED lights (blue violet, blue, and green red), could provide bright WLI, LCI, and BLI-LED as well as LASER endoscope, and a previous paper using this system with nonmagnification endoscope showed the efficacy of BLI and LCI for tumor detection and characterization regarding colorectal and gastric tumors [6, 16]. This system is less expensive than a laser endoscope and standard LED systems. Doctors in a private clinic commonly use this system in Japan because of its compactness and low cost. However, there have been no reports on the ability of this three LED light endoscope with BLI magnification for characterizing colorectal tumors. In the current study, we examined whether the tumor characterization of this three LED light BLI-LED technology was noninferior to that of BLI-LASER endoscopy.

## 2. Patients and Methods

**Figure 1:**
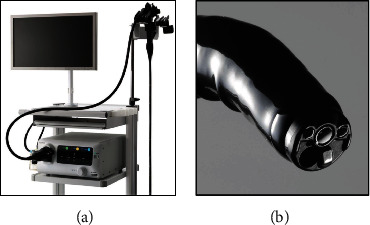
A compact light-processor combined LED endoscopic system and a dedicated LED endoscope. (a). A compact light-processor combined LED endoscopic system (EP-6000, Fujifilm Co.) and LED endoscope (6000 series; Fujifilm Co.). (b). The diameter of the LED endoscope (EC-6600ZP) is 11.7 mm, and the diameter of the working channel is 3.2 mm.

**Figure 2:**
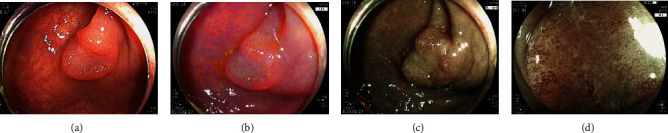
Case presentations of high-grade adenoma with an LED endoscope. (a) A nonpolypoid 10 mm lesion at the descending colon. Histopathology: high-grade adenoma. (b). Linked color imaging. (c). Blue light imaging without magnification. (d) BLI-LED, JNET Type 2B.

This observational study was conducted in the Department of Molecular Gastroenterology and Hepatology, Kyoto Prefectural University of Medicine. We retrospectively reviewed lesions that had been observed using both BLI-LED with an LED endoscopic system (EP-6000) and a magnified LED endoscope (EC-6600ZP) and BLI-LASER using a laser endoscopic system (LASEREO; Fujifilm Co.) and magnified LASER endoscopes (EC-L600ZP7 or EC-L600ZP) from August 2019 to March 2020 (Figures 1 and 2). In this study, patients undergo initial colonoscopy (LED or laser) before the treatment, such as endoscopic mucosal resection (EMR) and endoscopic submucosal dissection (ESD). Then, they underwent another colonoscopy within two months, which was not performed in the initial colonoscopy at the time of treatment. A total of 37 lesions were analyzed from 26 patients. In each of the 37 lesions, two magnified still images were extracted of the same area in the same tumor: one BLI-LED image and one BLI-LASER image.

The study outcomes were a comparison between BLI-LED and BLI-LASER regarding (1) diagnostic accuracy, (2) inter- and intraobserver assessments, and (3) detailed image comparison.

We analyzed the diagnostic accuracy of the two imaging types using the JNET classification system [17]. In the JNET classification, type 1 indicates hyperplastic polyps and sessile serrated lesions; type 2A, low-grade adenomas; type 2B, high-grade dysplasias and T1a cancers; and type 3, T1b cancers.

A total of 74 images were arranged at random by two expert endoscopists (K.I. and N.Y.) and were sent to the six endoscopists including three experts and three non-experts who were blinded. Then, two experts judged whether BLI-LED or BLI-LASER image was better according to their evaluations. Inter- and intraobserver agreement values for the assessment of the LED and LASER endoscopes were calculated. Kappa values were calculated for both expert and non-expert endoscopists to assess interobserver agreement. The same set of 74 pictures was randomly rearranged and sent to the endoscopists 4 weeks after their initial exposure to the pictures. The intraobserver agreement was then assessed. Two experts (K.I. and N.Y.) discussed and decided the JNET classification of lesions of this study.

For the detailed analysis of the diagnostic accuracy of LED and LASER images, two images—one each of LED and LASER—were arranged randomly side by side by two expert endoscopists (K.I. and N.Y.) and evaluated by the six endoscopists who were blinded. An original grading score based on the quality of visualization was applied: A score of 3 indicated that the LED image was better, 2 that the LED and LASER images were equivalent, and 1 that the LASER image was better (LED better/equivalent/LASER better).

**Figure 3:**
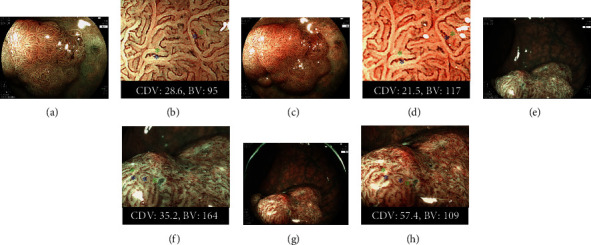
The comparison of Color difference value (CDV) and bright value (BV) between the BLI-LED and BLI-LASER endoscopic systems. (a). A nonpolypoid of 20 mm on the rectum. Histopathology: high-grade adenoma. BLI-LED: JNET Type 2B. (b) The CDV and BV for two points between the vessels (the red signs) and surrounding whitish area (the white signs) with BLI-LED were 28.6 and 95. (c) BLI-LASER of the same tumor: JNET Type 2A. (d) The CDV and BV were 21.5 and 117. (e) A nonpolypoid of 25 mm on the rectum. Histopathology: T1b cancer. BLI of LED: JNET Type 3. (f) The CDV and BV were 35.2 and 164. (g) BLI-LASER of the same tumor: JNET Type 3. (h) The CDV and BV were 57.4 and 109.

We compared the tumor characterization function of the BLI-LED system to that of the BLI-LASER system by calculating the color difference values (CDVs) of each of the 37 lesions. We used the CIEL∗a∗b∗ color space and delta ELab formulas to calculate the CDV (delta ELab), as described in a previous report [6, 16]. This value is used to evaluate whether an observer can detect a vessel in the color (differences can be detected clearly if the CDV is more than 5). In this CDV analysis, an endoscopist first determined two points on the vessel of the polyp which were exactly the same in both the BLI-LED and the BLI-LASER images (Figure 3). The CDV was then calculated between the vessels and the surrounding whitish area at those two points for both the BLI-LED and the BLI-LASER images. The mean value was set as the corresponding value for the polyp. Based on the CDV, we calculated the brightness value (BV) of the tumor vessel for the BLI-LED and the BLI-LASER using L∗ in the CIEL∗a∗b∗ color space and delta ELab formulas. The maximum value among these two values was set as the corresponding value for the polyp. We also analyzed each CDV according to polyp morphology and polyp histology related to the LED endoscope.

All images were obtained by two expert endoscopists (K.I. and N.Y.). All image preparation for this study was performed by two endoscopists, and all six endoscopists were blinded. Expert endoscopists were defined as endoscopists having conducted ≥5000 colonoscopes, including ≥2000 lesions of BLI-LASER/LED magnification. Non-experts endoscopists were defined as endoscopists having conducted <500 colonoscopes, including <200 lesions of BLI-LASER/LED magnification.

For bowel preparation, patients consumed a low-residue diet and took 10 mL on the day before the endoscopy. All patients received 1.0 L of highly concentrated polyethylene glycol solution with ascorbic acid (MoviPrep; Ajinomoto Pharma Co., LTD, Tokyo, Japan) on the morning of the day of the examination, as reported previously [18].

For lesion location, the right-sided colon was defined from the cecum to the transverse colon. The size of a polyp was defined by its maximum diameter and was calculated in accordance with the size of the snares. Morphologically flat polyps were diagnosed according to the Paris classification [19].

Tumor specimens were obtained by biopsy, polypectomy, and EMR. Thereafter, they were fixed with 10% formalin and histologically evaluated. Histopathological diagnosis was performed by three clinical pathologists according to the World Health Organization classification [20]. Intramucosal cancer was defined as high-grade dysplasia.

This research was performed with the approval of the Ethics Committee of Kyoto Prefectural University of Medicine (approval number, ERB-C-1600) and was carried out in accordance with the World Medical Association Declaration of Helsinki. And opt out was performed in this study.

## 3. Statistical Analyses

Quantitative data are summarized as mean, standard deviation, and range. When given together, the means and standard deviations are presented as means. Patient characteristics, content inspection, and detected lesions were compared using the Mann–Whitney *U* test, Wilcoxon signed-rank test, and chi-squared test. Inter- and intraobserver agreements were calculated using kappa statistics, where kappa = 0 demonstrated absence of agreement; <0.20, slight agreement; 0.21-0.40, fair agreement; 0.41-0.60, moderate agreement; 0.61-0.80, substantial agreement; and > 0.81, almost perfect agreement. Statistical significance was set at *p* < 0.05. All analyses were performed using SPSS Statistics (version 23.0; IBM Japan, Tokyo).

## 4. Results

The characteristics of 37 lesions from 26 patients were analyzed in this study. The mean polyp size (mean ± standard deviation) was 18.9 ± 13.5 mm, and there were 8 right-sided colon polyps (21.6%); 21 polyps (51.6%) were polypoid. Histopathology revealed 6 hyperplastic polyps, 7 sessile serrated adenoma and polyps, 9 low-grade adenomas, 12 high-grade dysplasias, 3 T1a cancers, and 1 T1b cancer (Table 1).

Using JNET classification for BLI-LED tumor characterization, the diagnostic accuracy of type 1 tumors was 100.0% (13/13) (Table 2). For type 2A, the diagnostic accuracy was 71.4%. For type 2B, the diagnostic accuracy was therefore 66.7%. For type 3, the diagnostic accuracy of 100.0%. Regarding BLI-LASER, the diagnostic accuracy of types 1, 2A, 2B, and 3 was 100.0%, 90.0%, 100.0%, and 100.0%, respectively. The overall diagnostic accuracy rates of BLI-LED and BLI-LASER of overall, experts, and non-experts were 89.6% and 88.3% (*p* = 0.65), 90.1% and 87.4% (*p* = 0.52), and 89.2% and 89.2% (*p* = 0.99), respectively.

The interobserver agreement was substantial (BLI-LED images:*κ* = 0.612, all; 0.610, experts; and 0.695, non-experts, respectively, and BLI-LASER images:*κ* = 0.622, all; 0.656, experts; and 0.731, non-experts, respectively). The intraobserver agreement was also substantial (BLI-LED images: *κ* = 0.745, all; 0.765, experts; and 0.713, non-experts, respectively, and BLI-LASER images:*κ* = 0.825, all; 0.869, experts; and 0.768, non-experts, respectively).

Regarding the analysis of the detailed comparison of the aurality of images of BLI-LED and BLI-LASER, the ratio of BLI-LED better/equivalent/BLI-LASER was 17%/62%/21% overall. These ratios were 16%/83%/1% for experts and 19%/58%/23% for non-experts (*p* < 0.001).

With the analysis of the CDVs and BVs, the CDVs for BLI-LED and BLI-LASER were 28.2 ± 11.9 and 29.5 ± 11.9, respectively (Table 3). The BVs for BLI-LED and BLI-LASER were 115.6 ± 30.6 and 120.1 ± 28.5, respectively. There were no significant differences in the CDVs and BVs of vessels between BLI-LED (*p* = 0.653) and BLI-LASER (*p* = 0.518).

## 5. Discussion

In the current pilot study, we examined for the first time the efficacy of magnified BLI-LED of the new compact three LED light endoscopic system (ELUXEO Lite) and compared it to that of the BLI-LASER system. We analyzed tumor characterization using magnified BLI-LED of the optimal magnified endoscope with the compact light source-processor combined LED endoscopic system using three LEDs and compared it to a BLI-LASER system because there are no papers about this 3 LED light system with magnified BLI observation at the moment, though lots of endoscopists in the world use this endoscopic system. The diagnostic accuracy of BLI-LED for colorectal lesions was noninferior to that of BLI-LASER, and the inter- and intraobserver assessments revealed a substantial agreement. A detailed comparison of BLI-LED and BLI-LASER indicated that 62% of lesions were equivalent in terms of image quality. Additionally, as objective indicators, there were no differences in CDVs and BVs between the BLI-LED and BLI-LASER systems.

Narrow-band imaging (NBI) is used more frequently worldwide compared to BLI. On the other hand, either BLI-LED or BLI-LASER has also been used in many countries, including the United States, Japan, as well as European and South American countries, among others. Many previous studies have reported the efficacy of BLI-LASER and NBI in colorectal tumor characterization [1, 2, 8, 21–25]. Our previous report demonstrated that the diagnostic accuracy of BLI-LASER with magnification for 314 colorectal polyps was 84.3% [1]. Some papers from Japan and Brazil showed that the accuracy of differential diagnosis between neoplastic and non-neoplastic diminutive polyps was 95.5% and 98.4%, respectively, using BLI-LASER with magnification [8, 21]. Our previous multicenter study highlighted that the diagnostic accuracies of BLI-LASER and NBI for 104 neoplastic lesions were 74.0% and 77.8%, respectively [2]. A recent study also reported that the diagnostic ability of the JNET classification for colorectal lesions with magnifying endoscopy with BLI-LASER was comparable to that of magnifying endoscopy with narrowband imaging [24].

However, few previous reports have described tumor characterization using a four-LED light endoscopic system (ELUXEO) [6, 10, 26–29]. We previously compared the four-LED light and LASER systems in terms of polyp detection and polyp characterization, describing a theory of each system. Our previous study evaluated the difference between the images produced by LED and LASER [6]. The image of LCI in LED endoscope was significantly brighter than that in LASER endoscope. We wrote it in the Discussion section. Several papers have demonstrated the efficacy of both systems have. However, no paper has compared the three-LED light system with the LASER system [29]. In a retrospective study with videos and still images, BLI-LED with a magnified LED endoscope was effective for colorectal polyp characterization using the BLI Adenoma Serrated International Classification (BASIC): The interobserver agreement was good for mucus for the polyp surface domain (alternative chance-correlated coefficient: AC 0.92 with, and 0.88 without, optical magnification, *p* = 0.002), for featureless pit appearance (AC 0.9 with, and 0.8 without, optical magnification, *p* < 0.001), for round/non-round pit appearance (AC 0.77 with, and 0.69 without, optical magnification, *p* = 0.02) descriptors, and for the vessel domain (AC 0.81–0.85, *p* = 0.02) [10]. In another retrospective study using still images, the accuracy, sensitivity, and negative predictive values of colorectal polyp histology improved from 87% to 94%, from 79% to 96%, and from 81% to 95%, respectively, by a specific training for BASIC [27]. In a prospective study, BLI-LED without magnification was accurate enough to predict histology, and the sensitivity of BLI for prediction of adenomatous histology was 92.68%, with a specificity and accuracy of 94.87% and 93.75%, respectively [26]. In a recent prospective randomized study, BLI-LED endoscopy was superior to high-definition white light for polyp characterization; the accuracy was significantly higher with BLI than with high-definition white light for colorectal polyps (92% vs. 84%, *p* = 0.011) [28].

We previously reported for this unique three-LED light system (ELUXEO Lite; EP-6000) and an endoscope without magnification (EC-6600R and EC-6600P, Fujifilm Co.) that the diagnostic accuracy of BLI-LED using a 2-mm close-distance observation function (77.0%) was slightly higher than that of BLI-LASER without magnification (65.6%, *p* = 0.16) [6]. Additionally, we reported that the efficacy of polyp visibility for WLI and LCI using the CDV of this system was inferior to that of the LASER endoscopic system. In the current study, we used this system, and the endoscope with optical magnification and visualization between BLI-LED and BLI-LASER was compared for both expert and non-expert endoscopists. Regarding diagnostic accuracy, our study shows similar overall accuracy between BLI-LED and BLI-LASER. However, in relation to JNET type 2A lesions, which corresponds to the most of adenomatous lesions, the accuracy was only 71.4% with BLI-LED, while the accuracy was 90% with BLI-LASER. Regarding this difference, the system of LED endoscope uses 3 LEDs different from another high-quality LED endoscopic system with four LED lights. This might affect the results, though the analysis of color difference value as objective indicators did not show the difference of them. Now, we are arranging a prospective study for examining the comparison of the four LED lights endoscopic system (ELUXEO) and the endoscopic LASER system. The overall intraobserver agreement for the three-LED light system was substantial, whereas the LASER system showed an excellent kappa value (*k* = 0.825). This also might be affected by the different contrast and color of the three-LED light system compared to the LASER endoscope. For experts, 83% of lesions were evaluated as equivalent. Only 58% of lesions were evaluated as equivalent for non-experts, and LASER images were better in 23% of lesions. This suggests that BLI-LASER is more suitable for non-experts because of the slightly higher contrast provided for the surface and vessel patterns. Now, we are arranging a prospective study for examining the comparison.

The cost of the compact three-LED light system in this study is low compared to both the LASER system with an endoscope and a four-LED light system (ELUXEO).

This study had several limitations. First, the study was conducted at a single center. Second, this study had a retrospective design. Third, the number of patients included in this study was small. We included only 37 lesions including only eight diminutive lesions, which were analyzed retrospectively by six endoscopists. We could not determine the efficacy of the three-LED light system in examining diminutive lesions. A bias might have exist in this study. Fourth, there might have been a selection bias for patients because only patients who received endoscopic treatment were enrolled. Fifth, in each of the 37 lesions, two magnified still images were extracted from the same area of a tumor. This extraction of images might induce a bias in the study, as the best images were selected. Sixth, two images of BLI-LED and BLI-LASER were not taken under perfectly similar condition about angle, air insufflation, and distance, and this might affect the results. A real-time video analysis should be performed in a future study. We should schedule a prospective and randomized study with a larger number of lesions to compare these two technologies in the future. Additionally, the comparison between BLI of this new three-LED light system and the widely available technology of magnified NBI should be performed.

In conclusion, we demonstrated, for the first time, the efficacy of tumor characterization for colorectal lesions using magnified BLI-LED of the optimal magnified endoscope with a compact combined light source-processor LED endoscopic system using three LEDs. The diagnostic accuracy and CD values of BLI-LED with the unique LED endoscope for colorectal lesions were noninferior to those of BLI-LASER.

## Figures and Tables

**Table 1:** Clinical characteristics of the 37 lesions observed with BLI-LED and BLI-LASER.

Number of cases	37
Number of patients	26
Age, mean ± SD; years	67.2 ± 13.9
Sex, % (*n*), male/female	50 : 50 (13 : 13)
Average size (range) (mm)	18.9 (2-70)
Location, % (*n*)	
Right/left/rectum	11.4/11.4/77.2 (8 : 8 : 21)
Morphology, % (*n*)	
Polypoid/nonpolypoid	56.8 : 43.2 (21/16)
Histopathology	
HP:SSL:LGA:HGD:T1-	16.2 : 18.9 : 24.3 : 32.4 : 8.2 (6 : 7 : 9 : 12 : 3)

BLI-LED: magnified blue light imaging, BLI-LASER: magnified blue laser imaging, SD: standard deviation, right: cecum to transverse colon, left: descending colon to sigmoid colon, HP: hyperplastic polyp, SSL: sessile serrated adenoma and polyp, LGA: low-grade adenoma, HGD: high-grade dysplasia, T1-: cancer invading deeper into the submucosal layer.

**Table 2:** The comparison of the tumor characteristics using JNET classification between BLI-LED and BLI-LASER.

JNET	Total *n* (%)	HP *n* (%)	SSL *n* (%)	LGA *n* (%)	HGD *n* (%)	T1- *n* (%)	Diagnostic accuracy Overall Experts Non-experts
BLI-LED							89.6∗ 90.1∗∗ 89.2∗∗∗
1	13 (100)	6 (46)	7 (54)	0	0	0	
2A	7 (100)	0	0	5 (71)	2 (29)	0	
2B	15 (100)	0	0	4 (27)	10 (66)	1 (7)	
3	2 (100)	0	0	0	0	2 (100)	

BLI-LASER							88.3+ 87.4++ 89.2+++
1	13 (100)	6 (46)	7 (54)	0	0	0	
2A	10 (100)	0	0	9 (90)	1 (10)	0	
2B	11 (100)	0	0	0	11 (100)	0	
3	3 (100)	0	0	0	0	3 (100)	

∗ vs. ^+^: *p* = 0.65, ∗∗ vs. ^++^: *p* = 0.52, ∗∗∗ vs. ^+++^: *p* = 0.99. JNET: Japanese narrow band imaging team classification, BLI-LED: magnified blue light imaging, BLI-LASER: magnified blue laser imaging, HP: hyperplastic polyp, SSL: sessile serrated lesions, LGA: low-grade adenoma, HGD: high-grade dysplasia, T1-: cancer invading deeper into the submucosal layer.

**Table 3:** The analysis of the CDV and BV for BLI-LED and BLI-LASER.

	BLI-LED	BLI-LASER	*p* value
CDV, mean ± SD	28.2 ± 11.9	29.5 ± 11.9	0.653
BV, mean ± SD	115.6 ± 30.6	120.1 ± 28.5	0.518

CDV: color difference value, BV: brightness value, BLI-LED: magnified blue light imaging, BLI-LASER: magnified blue laser imaging, SD: standard deviation.

## Data Availability

The patient data used to support the findings of this study are available from the corresponding author upon request. However, some of these data are restricted by the institutional review board of the Kyoto Prefectural University of Medicine.
